# Correction: On the relationship between Pathogenic Potential and Infective Inoculum

**DOI:** 10.1371/journal.ppat.1011191

**Published:** 2023-02-23

**Authors:** Daniel F. Q. Smith, Arturo Casadevall

References 47 and 48 are incorrect. Please see the correct reference below.

47. Wiederhold NP, Najvar LK, Bocanegra R, Kirkpatrick WR, Sorrell TC, Patterson TF. Limited Activity of Miltefosine in Murine Models of Cryptococcal Meningoencephalitis and Disseminated Cryptococcosis. Antimicrobial Agents and Chemotherapy. 2013;57: 745–750. doi:10.1128/AAC.01624-12

48. Wang JP, Lee CK, Akalin A, Finberg RW, Levitz SM. Contributions of the MyD88-Dependent Receptors IL-18R, IL-1R, and TLR9 to Host Defenses following Pulmonary Challenge with Cryptococcus neoformans. PLOS ONE. 2011;6: e26232. doi:10.1371/journal.pone.002623
