# Supplementary material for: On the relationship between Pathogenic Potential and Infective Inoculum
Source: PLoS Pathog. 2022 Jun 13;18(6):e1010484. doi: 10.1371/journal.ppat.1010484 (PMC9232127; doi:10.1371/journal.ppat.1010484)
Supplement: Attachment — Submitted filename: Pathogenic Potential Reviewer Response letter_051122.docx [file ppat.1010484.s003.docx]

May 11^th^, 2022

Michal A Olszewski, DVM, PhD, Guest Editor

Sarah Gaffen, Section Editor

Kasturi Haldar, Editor-in-Chief

Michael Malim, Editor-in-Chief

*PLOS Pathogens*

Re: Minor revisions for Manuscript PPATHOGENS-D-22-00571

Dear Drs. Olszewski, Gaffen, Haldar, and Malim,

We appreciate your consideration of our manuscript entitled, “On the relationship between Pathogenic Potential and Infective Inoculum,” (PPATHOGENS-D-22-00571) which we hereby resubmit for publication as an original research article in *PLOS Pathogens* following minor revisions*.*

We believe we have addressed most of the concerns of the reviewers and have included an itemized response to the constructive feedback and minor revisions suggested by the reviewers below. In summary, we have revised the manuscript to include better clarifications for our findings and reasoning behind terms used, such as description of why we have used mortality in the pathogenic potential equations. We also made edits to include more precise language to describe terms such as “Fs” which we have now defined as Fraction with sign or symptom. We have also separated the Discussion section into several sub-sections in order to enhance readability, as suggested by Reviewer 1.

We thank the reviewers and the editor for their enthusiasm for our work and their constructive comments, suggestions, and feedback. We believe that their suggestions have improved the clarity and preciseness of our manuscript and the suggested experiments have strengthened our previous findings.

Regards,

Daniel Smith

Arturo Casadevall

**Part I - Summary**
Please use this section to discuss strengths/weaknesses of study, novelty/significance, general execution and scholarship.

1. **Comment:** Reviewer #1: The study by Smith and Casadevall is an interesting approach towards developing a relationship between the pathogenic potential of a microorganism and its infective inoculum. Building on their previous work, the authors develop relationships for the pathogenic potential of fungi and bacteria in Galleria mellonella and compare the results to findings in literature. The result is some interesting quantitative insight in how different microbes cause pathogenicity. Few comments are below.

**Response:** Thank you for your interest in our work, kind words, feedback and suggestions!

2. **Comment:** Reviewer #2: The manuscript by Smith and Casadevall “On the Relationship between Pathogenic Potential and Infective Inoculum” is a refinement of previous papers on a mathematic model of pathogenic potential. In this iteration, the authors extend their studies to incorporate infective inoculum into their calculation.

   Overall, this paper adds to the literature of mathematical biology or mathematical microbiology if you will. The work draws upon previously published work as well as new data. The modeling is largely centered on moth larvae but does incorporate some mouse data. There are some concerns about interpretation and conduct of this work.

**Response:** We appreciate the constructive feedback and have made the appropriate edits to help assuage the concerns expressed in the review.

3. **Comment:** Reviewer #3: This manuscript is based on the premise that current quantitative measures of pathogenicity are poor, and the goal is to provide an improved quantitative measure. The authors have previously defined a concept called pathogenic potential that is an attempt provides a numerical measure of a pathogen’s virulence that is improved over LD50. The authors define pathogenic potential is a numerical description of an individual microbe, virus, or parasite’s ability to cause disease in a host using the independent variables of inoculum and host, and determining symptomology, mortality and time. Taking the concept of their 2017 paper, they now perform experiments to investigate the relationship between infective inoculum and pathogenic potential of Cryptococcus neoformans in larvae of Galleria moths. The authors establish a new parameter, the pathogen pathogenicity constant or Kpath to quantitatively compare the relative virulence and pathogenicity of a microbe in each host. I have reviewed the mathematic equations and cannot find an error. The authors have appropriately identified the limitations of the study. The authors define different relationships between PP, Fs and I and provide different explanations for the different relationships. The concepts are helpful defining potential host and virulence mechanisms. A lot of thought has been put into the interpretations provided in Table 1 and they are a powerful addition to the concept.
4. **Response:** Thank you for this detailed and supportive summary of our work and the constructive and helpful feedback throughout the following review

**Part II – Major Issues: Key Experiments Required for Acceptance**
Please use this section to detail the key new experiments or modifications of existing experiments that should be absolutely required to validate study conclusions.

Generally, there should be no more than 3 such required experiments or major modifications for a "Major Revision" recommendation. If more than 3 experiments are necessary to validate the study conclusions, then you are encouraged to recommend "Reject".

5. **Comment:** Reviewer #1: (No Response)

**Response:** No additional experiments were performed.

6. **Comment:** Reviewer #2: The authors in Figure 3 propose that one can calculate the smallest inoculum that causes pathogenicity. It would be useful if they would prove that their calculation from the y intercept is accurate.

**Response:** We considered doing such an experiment and in doing so realized that it was not trivial. We believe experimental proof of the lowest pathogenic inoculum is an interesting biological problem that is perhaps worth a study in its own right since the experimental approach would need to validate the meaning of negative results as the inoculum decreased. As the inoculum was progressively reduced, the death of infected larvae would approach the baseline death of uninfected larvae. Consequently, it would be difficult to discern naturally occurring death from infectious disease as we approach the lowest inoculum that caused disease. Another experimental limitation in this system is that the experimental observation time is limited by the larvae molting in moths. In addition, the experimenter would face the potential of increasing experimental error in the preparation of smaller inocula since the approach would still rely on dilutions of a concentrated suspension of microbe. These limitations are probably why the pathogenesis field adopted LD50s as a measure of virulence early in the 20^th^ century. Furthermore, as another reviewers noted (see point 11 below) the LPI may be highly dependent on the growth state of the microbe and growth state are notoriously difficult to control. The combination of these effects is that the system would become increasingly noisier as the lowest pathogenic inoculum was approached requiring ever increasing numbers of larvae to finds signals. Hence, we felt that it was topic was sufficiently complex to be beyond this study. However, the reviewer comment has piqued our interest in this problem, and we hope to do some preliminary explorations at smaller inoculum to see how things behave and what is possible. Stay tuned!

7. **Comment:** Reviewer #3: none

**Response:** No additional experiments were performed.

**Part III – Minor Issues: Editorial and Data Presentation Modifications**
Please use this section for editorial suggestions as well as relatively minor modifications of existing data that would enhance clarity.

8. **Comment:** Reviewer #1: 1. The Discussion is somewhat dense. It would be easier for the reader if the authors could parse out the discussion into several sections. Also, the authors’ logic in referring to Feynman’s terminology in line 305 is unclear.

**Response:** We have made some slight edits to the section describing Feynman’s description of seeking new laws. We included this section to describe why we undertook this project to experimentally prove, disprove, revise, or add nuance to a possible natural law of pathogenic potential following the previous publication of the equations used. At our end we found Feynman’s words very helpful and reassuring. His observation that natural laws and mathematical relationships are first thought of by human minds, and then validated by experimental work, helped us understand the course of our own work. We have also broken up the discussion into several sub-headings that should help break up the density of the discussion.

9. **Comment:** Furthermore, the authors should use the word ‘equation’ instead of ‘formalism’ when referring to equations in lines 60 and 72. Similarly, the authors should not use the word ‘simulate’ in line 247 and Figure 9, as that is commonly used when a computer simulation is performed (which does not appear to be the case here).

**Response:** Thank you for this helpful advice. We have replaced the term “formalism” with “equation” throughout the text (Lines 60, 72, 74 307, 448). We have also changed the phrase “simulated”/”simulating” to “modeled”/”modeling” (lines 251, 253, Figure 9).

10. **Comment:** 2. It appears that the end goal for such a model is to utilize it for hosts with adaptive immunity or in examining drug efficacy. The authors have shown data comparing Fs/T vs. I values for Galleria vs. mouse. Have they tried to examine (or can they comment on) how these plots change when the host is receiving a drug post-infection?

**Response:** Since treatment with an antimicrobial drug is expected to reduce Fs and mortality while the starting inoculum in the system remains the same, such therapy is expected to reduce the PP and PPT values. The use of PP and PPT values to measure antimicrobial agent or vaccine efficacy (or measuring the effect of these therapeutics on PP or PPT) has been previously commented on in the recent paper describing the PPT equations (Casadevall, 2022). We have included a sentence in the discussion to comment on how PP is affected by antimicrobial drugs. This sentence reads, “Measures of Pathogenic Potential would also change following administration of an effective antimicrobial therapeutic, where the expected Fs and M values decrease, T increases, and I remains constant, and as such, changes in PP and PP _T_ following treatment can be used to measure therapeutic efficacy. Conversely, immunosuppressive treatments or conditions that broadly enhance host susceptibility to infection would lead to increased PP and PP _T_ , which can then be used to measure infection-related the risks involved in certain treatments.”

11. **Comment:** 3. The calculation of LPI (line 141), and especially the comparison between intravenous and intranasal is particularly interesting. Have the authors considered the possibility of LPI depending on the stage of growth? For example, microbes in exponential phase are more likely to be lethal than those in the stationary phase. Most studies utilize unsynchronized cultures, making this difficult to estimate, but the answer could be insightful.

**Response:** We agree with the reviewer that grow state is likely to be very important but suspect that its effects magnify as the inoculum is reduced and each microbe makes a greater contribution to the outcome. For our experiments, the *C. neoformans* and *C. albicans* cells are grown to stationary phase in a nutrient-rich media, which helped standardize infective preparations between experiments. While we think the answer to this would be interesting and provide useful insight into how the metabolic state of the microbe affects pathogenicity (including culture growth at different temperatures, and in media of different nutrient content), we believe that is beyond the scope of the current work. (see also response 6 above)

12. **Comment:** 4. The mathematical model is quite simple and preliminary. There are no issues with it, it's just very simple. Most likely, this model would need to be modified once the authors go to a host with adaptive immunity. Beside this, it is not the first time the authors have raised the topic of pathogenic potential, thus the novelty of this paper is somehow decreased.

**Response:** We agree the models proposed are preliminary and pending further investigation in other host organisms. In this work, we sought to use a readily available and relatively straightforward model for studying virulence without the additional complexities of the adaptive immune response and effect of various host strains and inbreeding, as is the case with mice. However, since there appear to be many overlaps in microbial pathogenesis in *G. mellonella* and mammals and as seen in Figure 4, we would anticipate some degree of applicability for the model in more complex hosts. Although the pathogenic potential complex was described earlier this develops the topic further and provides the first experimental exploration of the topic, an effort that revealed unknown differences between different pathogens.

13. **Comment:** Reviewer #2: 1. It is unclear how the authors calculate an Fs for a moth larva. In the original manuscript in mSphere the definition of fraction symptomatic acknowledged that there is a range. How the authors calculated an Fs for a moth needs to be described.

**Response:** For this study, we calculated Fs based on the observed or reported mortality of larvae, as more detailed reports of signs and symptoms within *G. mellonella* larvae is not found consistently throughout literature. We have edited the manuscript to include description of how we defined Fs in the methods section. We also added sentences in the Discussion to discuss various ways that Fs can be calculated in the *G. mellonella* model including measuring melanization or reduction of movement.

14. **Comment:** 2. The data with dimorphic fungi is difficult to interpret and hard to believe that the higher inoculum is less pathogenic so to speak. I think if they pore over the data with mice that they will find that is not the case and that the calculations using moths may not be accurate. Moreover, the temperatures used to study dimorphs might cause stress in moths and that is not discussed.

**Response:** We too were surprised by those result with pathogenic fungi. We agree that the data for these experiments in larvae is counterintuitive and likely to not be applicable to mammalian models but kept the analysis in the paper to provide examples of unexpected results and to avoid cherry-picking datasets. We have included some analysis from literature studying the dose-dependency of mortality in mice infected with *H. capsulatum* and *P. brasiliensis* as Supplementary Figure 1. We have also added that to the results and discussion section respectively:

*“Dissimilar to the findings in G. mellonella, analysis of murine infection with P. brasiliensis reveals an inoculum-dependency for PP, PP_T_, and Fs/T similar to what is seen with other fungi (Supplementary Figure 1A-D). There is no clear inoculum-dependent effect on PP or PP_T_ in H. capsulatum infection of mice, and there is a roughly positive linear relationship between Fs/T versus I (Supplementary Figure 1E-H), which is unlike other fungal Fs/T vs. I relationships observed in G. mellonella. These data underscore the need to compare PP calculations within the same host organism and illustrates that microbes may cause disease in different ways in different hosts.”*

*Additionally, data extracted from mouse literature indicate there is a positive Fs/T vs. I relationship and a positive kpath value for P. brasiliensis and H. capsulatum. This underscores the importance of comparing data within the same host, and the possibility that the same microbe could have different mechanisms of causing to disease in different hosts. This can then in turn affects the relationship between PP, PPt, and Fs/T versus I relationships.*

We changed the following sentences in the results section discussing the data from *H. capsulatum* and *Paracoccidioides spp.:*

*“Future studies may want to further investigate the mechanism underlying the unique dose-dependency, or independency, of H. capsulatum and P. lutzii infections in G. mellonella, or include quantification of the reported dose-dependent melanization response in larvae, which could be used as the Fs value and provide more nuanced and intuitive inoculum dose-dependency in PP, PP_T_, and Fs/T.”*

*“Additionally, the higher temperatures for the 37˚C conditions may cause thermal stress on the larvae that could impact their immune response and baseline longevity (16,17).”*

15. **Comment:** 3. One factor that is missing is the doubling time of organisms and how that fits into the equation. We know from human observation that the tempo of disease in humans is often correlated with the doubling time of the organism. For example, pneumococcus causes rapid onset of symptoms whereas Mycobacterium tuberculosis is more insidious. The doubling times of these two bacteria differ substantially. It seems to me to a variable that needs to be acknowledged since the authors do incorporate survival time in their calculations. Perhaps for vertebrates, survival time is not the correct T but time to onset of symptoms.

**Response:** Thank you for the suggestions, and we do think the doubling time of the microbe is important in our understanding of pathogenic potential and its relationship with inoculum. While we haven’t incorporated doubling time directly into the equations, we believe that the doubling time is associated with the value of *kPath*, particularly for the microbes that tend to cause disease through persistence and growth within the host. We briefly mention this as a potential reason behind the kPath ~0 for the *H. capsulatum* and *P. lutzii* which exhibit remarkably slow growth in culture compared to *C. neoformans* and *C. albicans.* The sentence reads, “The general dose-independent effect on survival could be the result of the slow and irregular growth of the microbe (11–13), or a damaging immune response that kills the host in response to few or many microbes (Table 1). In this regard, *P. lutzii, P. brasiliensis,* and *H. capsulatum* are both slow growing fungi with doubling rates in media ranging from 13 to 21 hours (11–13), compared with the ~2 hour doubling time of *C. neoformans* in culture (14) and ~5 hours in vivo during infection of *G. mellonella* hosts (15).” One reason we do not include microbial doubling time in the equations is that we do not know the precise doubling time *in vivo,* which may be significantly slower than the doubling time in culture and highly dependent on host, organ specificity, and inoculum culture conditions. Furthermore, doubling time may change as a function of the immune response, which would kill some of the replicating microbes to reduce the rate at which microbial burden accumulates. We added this additional sentence in the discussion to reiterate the potential importance of microbial doubling time in PP/kpath determination: “Additionally, for microbes that cause disease through growth and persistence within tissues, a high *k_Path_* could be associated with fast microbial doubling times, whereas low or zero *k_Path_* could be associated with microbes that have slower rates of growth within the host.”

Regarding using symptom onset time as T instead of median survival time, we believe that could be useful for future iterations of PP equations that deal primarily with human patients, where time of symptom onset could be more reliably determined. T as time until symptom onset rather than mortality could be useful in understanding disease in humans because, in ideal scenarios, people receive treatments and survive infection, which would confound the median survival time measurements.

16. **Comment:** Reviewer #3: 1. The concept of Kpath (slope of I vs Fs/T) is elegant because of its simplicity. However, by introducing the concept of mortality in addition to symptoms and signs the concept has become more complex and lost some of its elegance. As I will describe below, mortality is a sign of disease and does not need to be a unique variable. The authors either need to do a better job of explaining why it is necessary to incorporate this additional level of complexity or remove it.

**Response:** Thank you for your appreciation of the Kpath equation, and we appreciate the constructive feedback regarding morality as a sign/symptom of disease versus as separate variable. In the previously published equations for PP and PP_T,_ mortality was added as a variable in order to adjust pathogenic potential for microbes that have high mortality versus low mortality. The inclusion of the 10^M^ term allows for “amplification” of the PP for lethal microbes or viruses. It was added in the original formulations because mortality is an extreme event that suggested different pathogenic interactions where the host received irrecoverable amounts of damage. Without the inclusion of the mortality term, a cold virus that causes 0% mortality but mild symptoms in 100% of individuals at an inoculum of 10^3^ virions would have the same PP as a hypothetical hemorrhagic fever virus that causes 75% mortality and symptoms in 100% of individuals at an inoculum of 10^3^ virions. We have added a sentence in the introduction that now states, “Mortality, as the 10^M^ term, was included as a separate variable in order to amplify the pathogenic potential of lethal microbes versus non-lethal ones.” A reference was provided to the original 2017 work (Casadevall, 2017).

17. **Comment:** 2. The authors use the word “symptom”. From JAMA network: “A symptom is a manifestation of disease apparent to the patient himself, while a sign is a manifestation of disease that the physician perceives. The sign is objective evidence of disease; a symptom, subjective.” Since the moths are not reporting their subjective impression of how they feel, moths have “signs” not symptoms. I encourage the authors to define the term Fs as fraction with signs or symptoms.

**Response:** Great point! Thank you for the clarification of “symptom” versus “sign.” We have changed the definition of Fs to be “Fraction with signs or symptoms,” and have changed all other uses of “symptom” or “symptomatic” to “signs or symptoms” or “with signs of disease.”

18. **Comment:** 3. While the authors present an appropriately simple model to test the concept, an important strength is the ability to extend it to all microbes and disease manifestations. Consequently, Fs should be defined by what is most appropriate for the microbe and host. By including mortality, the concept is made more complex and also limiting in its ability to be used in other context. By way of an example, it would be powerful to use the concept to assess pathogenic potential of different strains and species of enteropathogenic bacteria with Fs as manifestations of enterocolitis.

**Response:** Again, great point! As the reviewing deduced, our emphasis on mortality is simply a function of working with *Galleria* where this was the measured outcome. We do not believe the inclusion of the mortality term is limiting the applicability of the findings but recognize that other measures would be needed in other systems. As stated above, the inclusion of mortality allows for differentiation between lethal and non-lethal microbes or viruses that may cause similar proportion of disease. The Fs term should be used for the appropriate microbe or host being studied. As for your example, signs or symptoms for enteropathogenic bacteria and viruses in a human host would be recorded as presence of enterocolitis (fever, nausea, diarrhea, etc), while Fs for influenza in mice would likely be recorded by weight loss or hypothermia, and Fs of *G. mellonella* infected with fungus would be death or systemic melanization. Hence, we agree with the reviewer and have added a sentence borrowing some of her/his words in the discussion: “*In applying these concepts, Fs should be defined by what is most appropriate for the microbe and host depending on the measurable outcomes of the host-microbe interaction.”*

19. **Comment:** 4. Fs is defined in each experiment. In the current experiments the authors used the fraction that has died as the measure of Fs, which is appropriate for these experiments. However, death may not be the most appropriate measure of pathogenicity for different types of microbes. In experiments where another parameter might be used as the sign, that parameter could be used to determine Fs. I would encourage the authors to use Fs and define the symptom or sign (which would include death) or explain why it is necessary to increase the complexity by using a combination of Fs and mortality in pathogens where it is inappropriate to do so.

**Response**: Certain microbes in *G. mellonella* triggers the melanin-based immune response or triggers behavioral changes in the larvae that could be recorded as a quantifiable sign of disease that could then be used to determine the Fs term. However, this is not consistently reported in literature in a way that would be useful for calculating Fs (i.e., unreported or only reported in representative images that do not contain reliably quantifiable data.), thus for the data analyzed, we used mortality as a sign of disease (Fs) because that would be the most standardized parameter we could use. We have included a section in our discussion that reads, *“For the purposes of this work, we calculated the Fs value as equivalent to the mortality of the larvae due to the consistency of mortality being reported in literature. G. mellonella can exhibit signs and symptoms of infection, including systemic melanization and reduction in movement, which could be used to calculate a Fs value independent of morality. These values are occasionally reported, but more widespread reporting of signs and symptoms of infection could be helpful in providing more nuanced calculations in the future.”* We have also included the following sentence in or methods section, “*For the purposes of this paper, Fs value were calculated as the total mortality of larvae or mice since cumulative incidence for the signs or symptoms of infections in G. mellonella and mice are often unreported or inconsistent in literature.”*

20. **Comment:** 5. The authors have added a mortality term. The implication is that mortality correlates with a more pathogenic microbe. Death is a complex pathologic process, and while it may be a philosophical point, there are many things that are worse than death. I would encourage the use of the most appropriate Fs for each disease and not to include mortality or explain why it is necessary to increase the complexity by using a combination of Fs and mortality.

**Response**: Thank you for your feedback. As we have written above, we have included a description of why mortality is included. We have also edited the introduction to define Fs as “fraction of the population with microbe-relevant signs or symptoms.”

21. **Comment:** 6. Authors provide mathematical descriptions for positive logarithmic and negative exponential relationships. The manuscript would be stronger if an intuitive interpretation were also provided.

**Response**: These intuitive explanations were included in the discussion and Table 1, but we have added underlines to the common interpretation of the results in Table 1, and we have added these sentences following the introduction of the concept of “negative exponential,” “positive logarithmic,” and “positive linear.”:

*The negative exponential relationship between pathogenic potential and inoculum shows that the average microbe during an infection with high inoculum has smaller contribution to pathogenic potential and disease compared to the average microbe during a lower inoculum infection.*

*Since this relationship between inoculum and disease is logarithmic and not linear , it implies that microbes the higher inoculum on average contributes less to disease. This would be consistent with a microbe that causes disease from high microbial burden in the host due to exponential doubling of microbes.*

*The positive linear relationship between Fs/T and inoculum indicates that microbes contribute equally to disease during low and high inoculum infections, meaning that each bacterium a set contribution to disease. This is expected with microbes that secrete set amounts of toxins or inflammatory molecules that work in a dose dependent manner.*

22. **Comment:** 7. I would encourage the authors to compare the implications of different relationships. As is, the reader is forced to compare the interpretations in table 1 and to try to determine the differences.

**Response:** Thank you for the suggestion. These points were included in the discussion but we agree that they can be made clearer. We have edited the discussion to now be under several subheadings, which should help highlight the implications and comparisons of the different relationship. In addition, we have amended one of the sentences in the conclusion paragraph to read:

*For example, if a microbe causes damage through growth and survival, the order of magnitude (log) inoculum would likely be the relevant determining factor of disease (i.e. logarithmic Fs/T vs. I relationship), whereas if the microbe causes damage through toxins or lytic proteins, pathogenicity would likely be directly dependent upon each microbial cell (i.e. positive linear Fs/T vs. I relationship) (Summarized in Figure 10).*

23. **Comment:** 8. Figure 1c is described in the results section, but I was unable to find the figure.

**Response:** Thank you for the careful reading and pointing this out. The correct figure reference is Figure 1B, and the text has been corrected to reflect that.

24. **Comment:** 9. Line 316 “i” should be “I”.

**Response:** “i” was mistakenly left following a previous edit. It has now been removed.
